# Can sports events improve residents’ psychic income?

**DOI:** 10.3389/fpsyg.2022.938905

**Published:** 2022-07-19

**Authors:** Jie Cai

**Affiliations:** School of Sports, Shandong University of Finance and Economics, Jinan, China

**Keywords:** sports events, social capital, social identity, psychic income, residents

## Abstract

To better understand the positive impact of sports events on host cities, this study uses social capital and social identity theories combined with structural equation modeling (SEM) to explain the psychic income that sports events may bring to residents. The investigation and analysis of the Qingdao Marathon in 2021 show that (1) sports events can generate social capital; (2) social capital can positively influence social identity; and (3) social identity can significantly impact event excitement, city attachment, and city pride in psychic income.

## Introduction

Sports events, especially marathons, are thriving in China, and their economic benefits and social influence are widely concerned. Many event organizers expect higher benefits by providing high-quality products and services (Inoue and Havard, 2014). Sports events are often seen as catalysts for economic development because they raise the image and visibility of the host city (Cornelissen and Swart, 2010). The marathon links participants with the host city, forming a benign interaction to improve the image of the city (Smith, 2002). From the perspective of intangible assets, sports events can bring positive spillover effects and enhance civic pride (Baade and Matheson, 2016). Moreover, hosting sports events can improve local infrastructure, thus facilitating local development (Crompton, 2004). As one of the emerging events in China, the marathon meets people’s demand for sports experience. In addition, it is a social symbol that satisfies the cultural identity of individuals or groups. Therefore, we should pay attention to the invisible impact of marathon events on the residents of the host city.

Hosting sports events can bring social capital changes, especially in the overall city benefits (Philip Stone and Richard Sharpley, 2011). In terms of event impact, Preuss (Preuss, 2007) proposed the concept of “event legacy” to measure the lasting impact of sports events and argued that unintended and intentional impacts on cities beyond sports events constituted event legacy. Hosting sports events can greatly enrich the host city and provide intangible social benefits to its residents. To transform the psychic income of sports events from temporary entertainment to lasting social resources, city managers should use the social capital brought by sports events in order to establish the social identity of residents (Clopton and Finch, 2011). Sports events gather citizens together through activities that promote social interaction (enhance social capital), experience the honor of the city (deepen social identity), and enhance city pride (increase psychic income; Kaplanidoua, 2012).

Social capital and social identity can establish a foothold for social institutions or activities among the group members (Katz and Clopton, 2014). Some scholars have proposed that these two theories can be used to explain the continuous psychic income of sports events to group members (Clopton and Finch, 2011; Seifried and Clopton, 2013). Previous surveys have suggested that sports events are short-lived and that the excitement generated by them is hard to sustain (Li et al., 2013) and cannot be translated into sustained benefits (Mills and Rosentraub, 2013), lacking long-term economic returns (Barajas et al., 2016). Social capital and social identity provide a new perspective for studying sports events. Based on this, we can understand the role of sports events in the development of the psychic income of residents in host cities. Therefore, this study attempts to explore whether sports events can improve the psychic income of residents in host cities without considering economic benefits. Then, the impacts of sports events on the social capital, social identity, and psychic income of residents in the host city are analyzed.

## Theoretical basis and research hypothesis

### Sports events and social capital

Sports events can enhance the social capital of residents (Johnson et al., 2009). Bourdieu (1986) defined social capital as “the sum of actual or potential resources linked to a durable network of people,” which concentrated social capital through groups of homogeneous individuals (such as large sports events) and strengthened groups through communication between individuals, thus raising social capital. Graaff and Boulding (1951) observed a financial capital store necessary for continued existence in the economic unit, while in human nature, there should also be a psychic capital reserve that is vital for the mental health of individuals and society. Psychic capital can construct a reserve by providing a link consisting of positive feelings shared between individuals and larger groups. Boulding also proposed that a coherent body of thoughts, memories, and emotions may be shared between individuals and collective minds as shared psychic capital. Moreover, the lack of psychic capital may lead to collective depression and suicide. According to the relational dimension, social capital can be divided into bonding social capital and bridging social capital (Boggs, 2001). The characteristics of bonding social capital are that individuals with high similarity of social background are closely related to each other, enjoy small groups, and exclude other groups. The characteristics of bridging social capital are that individuals exchange more information and can provide each other with new information but lack emotional communication and social support.

Sports events or sports festivals serve as magnets for residents to gather in central locations and strengthen ties between members of society (Downward and Rasciute, 2011). Sports events can gather residents together, strengthen communication among them, especially those with the same social background, and promote the process of social capital (Johnson et al., 2006). In particular, the urban marathon is characterized by centralized display, extensive participation, competition in the same field, high integration, open cooperation, and social and cultural integration (Itsubo et al., 2009), thus directly impacting the social capital of residents. Sports events create social capital by providing opportunities for communication between participants, organizers, and visitors (Peachey et al., 2015a). In addition, sports events can catalyze residents to gather, interact, and identify with each other and provide opportunities to develop collective consciousness and improve relationships, thereby enhancing the social capital of residents (Schulenkorf, 2013). Hence, hypotheses are established as follows:

H1a: Sports events positively impact bonding social capital.

H1b: Sports events positively impact bridging social capital.

### Social capital and social identity

Putnam (2000) has deepened the understanding of social capital by describing it as the benefits that come from and contribute to society and social networks. The role of trust and reciprocity norms is highlighted in the evaluation of social network quality and individual choice, leading to a reflection on social identity. In a sports environment, social capital can provide opportunities for citizens to participate and for communities to develop, thereby enhancing community cohesion and social inclusion. When individuals are allowed to participate in social interaction, sports events positively impact their wellbeing (Sherry et al., 2015). In sports events, participants’ perception of their own importance allows the event to “inject new energy into the public atmosphere,” which breaks down the barriers of communication and society, provides a unique opportunity to explore controversial social issues, and deepens team identity (Fredline, 2004). Sports events provide the motivation for achieving social change and reciprocity, developing social capital, and deepening self-identity (Peachey et al., 2015b). Similar results were found in physical activity-based service-learning programs, where participants increased social capital through forming a public network, and the group and city identity of participants were promoted (Bruening et al., 2015). With many participants, the marathon plays an important role in developing social capital and enhancing social identity. Thus, hypotheses are established as follows:

H2a: Bonding social capital positively impacts team identity.

H2b: Bonding social capital positively impacts city identity.

H3a: Bridging social capital positively impacts team identity.

H3b: Bridging social capital positively impacts city identity.

### Social identity and psychic income

In sports, psychic income refers to the emotional and psychological benefits that individuals can derive even if they do not personally participate in a sporting event (Crompton, 1995). Psychic income from sports events usually includes city pride from increased visibility, civic pride from hosting large-scale events, enhanced collective self-esteem, and emotional investment in sports activities (Kim and Walker, 2012). Kim et al. (2015) measured the intangible effects of Super Bowl XLIII. They proposed that social identity can directly impact psychic income that is mainly composed of event excitement, pride, and attachment. Team billboards displayed in public places or flags hanging in stadiums can instill the social identity of individuals to enhance their sense of belonging (Heere et al., 2013). The glory brought by sports events and the attraction to tourists from other cities can make local residents feel different. The popularity of sports events can enhance the social identity of residents and increase their psychic income (Shapiro et al., 2012). By analyzing the impact of the 2010 Football World Cup in South Africa, it is found that social identity can provide opportunities to increase psychic income (Gibson et al., 2014). Therefore, hypotheses are proposed as follows:

H4a: Team identity positively impacts event excitement.

H4b: Team identity positively impacts city attachment.

H4c: Team identity positively impacts city pride.

H5a: City identity positively impacts event excitement.

H5b: City identity positively impacts city attachment.

H5c: City identity positively impacts city pride.

## Materials and methods

### Research questions

**FIGURE 1:**
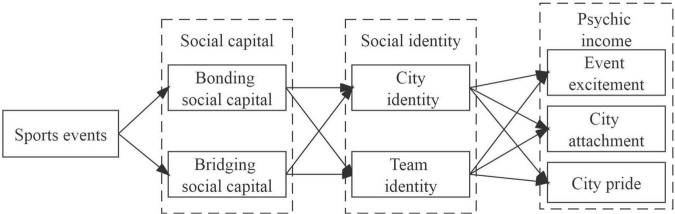
Research model.

Quantitative research was conducted using questionnaires. The general objective of the research was to investigate the attitudes and behaviors of Qingdao residents toward the Qingdao Sports Bureau (2021). Based on the objective, this study established a research model of social capital, social identity, and psychic income of residents in the host city after sports events (Figure 1).

### Research tool and data collection

The questionnaire used a seven-point Likert scale from “1” to “7” for “strongly disagree” to “strongly agree.” In addition to the basic information of the respondents, the questionnaire also involved sports events, social capital, social identity, and psychic income.

Measurement items of sports events were adapted from Kaplanidou and Vogt (2007). The content included “The race track and scenery along the way are satisfactory” and “The race is well organized with reasonable rules.” Social capital included two indicators, namely bonding social capital and bridging social capital, which were capital subscales of Williams’ Network Social Capital Scale (Dmitri, 2017), including items such as “Residents in Qingdao are trustworthy” and “Residents in Qingdao are hospitable.” Social identity included two indicators, namely city identity and team identity. For city identity, we adopted Luhtanen and Crocker’s collective self-esteem scale (Luhtanen and Crocker, 1992) and some contents of Mael and Ashforth on organization identification (Mael and Ashforth, 1992), such as “I am very interested in what others think about Qingdao” and “The success of Qingdao is the success of Qingdao residents.” Team identity was measured by Wann and Branscombe’s (1993) Sports Audience Identity Scale, with adapted questions such as “I hope the athletes of the Qingdao marathon team win” and “I am very concerned about the performance of athletes from Qingdao.” The psychic income included three indicators, event excitement (evex), city attachment (att), and city pride (pride). The revised questions according to Kim and Walker’s (2012) psychic income scale included “This year’s marathon brings joy to Qingdao residents,” “Qingdao will gain a positive image as the host city of this marathon,” and “This marathon deepens my affection for Qingdao.”

### Sampling and participants

Data collection began 1 month after the end of the Qingdao Marathon. Before the mass distribution of the questionnaire, 15 Qingdao residents were invited for a pre-test and one-to-one interview. The overly academic sentences were modified to ensure that the items were easy to understand. Finally, the samples were drawn from some respondents in a wider community (Tharenou et al., 2007), and the target population was Qingdao residents. This study was approved by the local Ethics Committee (2021/002, 17 June 2021) and was conducted following the Declaration of Helsinki established by the World Medical Association. Submission of the online survey after completion implied consent to participate in this study, which was declared to respondents in the beginning of the survey. A total of 408 questionnaires were sent out, of which 372 were valid. The effective recovery rate was 87.12%, and the ratio of the item to sample size was 1:15.5. Based on the sample data size, this study reached the optimum requirements for structural equation modeling (SEM; Sharma and Crossler, 2014). Table 1 shows the answers of the respondents regarding their demographic characteristics.

**TABLE 1:** Demographic characteristics of valid samples (*N* = 372).

Variable		Percentage (%)
Gender	Male	32.5
	Female	67.5
Age	18–25	23.1
	26–40	40.1
	41–55	28.2
	Over 56	8.6
Educational level	High school	23.9
	Bachelor degree	48.4
	Master	24.2
	PhD	3.5
Profession	Student	5.1
	Private employee	47.8
	Freelancer/business owner	26.1
	Employee in the public sector	8.9
	Unemployed	12.1
Time lived in Qingdao	Under 1 year	2.2
	1–5 years	10.8
	6–10 years	37.0
	11–15 years	42.7
	Above 15 years	7.3

## Results

The measurement model and structural model were assessed using SEM. We used SEM because its flexibility allowed us to model the proposed relationships between several predictors and criterion variables that were based on unobservable latent variables (Chin, 1998). Latent variables were connected to observable variables by a measurement model. Additionally, SEM allows the analysis of the dependencies of latent variables, offering the opportunity to analyze the relationships among the psychological constructs without measurement errors (Nachtigall et al., 2003).

### Reliability and validity analysis

Table 2 summarizes the assessment results of the measurement model. The results supported construct reliability and the convergent and discriminant validity of all constructs. Cronbach’s alpha (ranged from 0.811 to 0.922) indicated good internal consistency of the items of constructs. The composite reliability of constructs (ranged from 0.849 to 0.912) supported their validity. Convergent validity was assessed using the average variance extracted from the constructs (ranged from 0.652 to 0.776). Squared multiple correlations (ranged from 0.561 to 0.906) greater than 0.36 indicated good reliability of the items. Factor loadings ranged from 0.749 to 0.926 and were significant at 0.001. Table 3 shows the results of the discriminant validity test for each latent variable. The square root of the AVE value for most latent variables was greater than the absolute value of the correlation coefficient between these variables and other latent variables, reflecting the higher discriminant validity among the variables incorporated for analysis.

**TABLE 2:** Load amount of variable factor and reliability and validity index.

Latent variable		Estimate	SMC	CR	AVE	Cronbach’α
Sports events (sp)	sp1	0.881	0.776	0.876	0.702	0.811
	sp2	0.853	0.728			
	sp3	0.777	0.604			
Bonding social capital (bond)	bond1	0.880	0.774	0.912	0.776	0.868
	bond2	0.952	0.906			
	bond3	0.805	0.648			
Bridging social capital (bridge)	bridge1	0.784	0.615	0.882	0.714	0.922
	bridge2	0.865	0.748			
	bridge3	0.882	0.778			
Team identity (team)	team1	0.821	0.674	0.899	0.749	0.866
	team2	0.906	0.821			
	team3	0.867	0.752			
City identity (city)	city1	0.878	0.771	0.889	0.730	0.881
	city2	0.749	0.561			
	city3	0.926	0.857			
Event excitement (evex)	evex1	0.816	0.666	0.853	0.660	0.877
	evex2	0.837	0.701			
	evex3	0.783	0.613			
City attachment (att)	att1	0.837	0.701	0.849	0.652	0.873
	att2	0.766	0.587			
	att3	0.818	0.669			
City pride (pride)	pride1	0.874	0.764	0.877	0.704	0.890
	pride2	0.851	0.724			
	pride3	0.790	0.624			

**TABLE 3:** Test table of variable discriminant validity.

	AVE	sp	bond	bridge	team	city	evex	att	pride
sp	0.702	**0.838**							
bond	0.776	0.191	**0.881**						
bridge	0.714	0.120	0.023	**0.845**					
team	0.749	0.132	0.423	0.455	**0.865**				
city	0.730	0.077	0.256	0.250	0.217	**0.854**			
evex	0.660	0.070	0.225	0.238	0.466	0.263	**0.812**		
att	0.652	0.063	0.204	0.214	0.404	0.279	0.417	**0.807**	
pride	0.704	0.079	0.254	0.268	0.519	0.313	0.345	0.294	**0.839**

Bold values represent the square root of AVE (Average Variance Extracted) values.

### Hypothesis testing

Table 4 shows the results of the predictive accuracy assessment of the model. According to the fitting index of SEM, the RMSEA value is less than 0.08. In addition, CFI and TLI are greater than 0.9, χ2/DF is less than 3, and all fitting indexes reach the test standard, indicating that the model basically meets the fitting requirements and possesses appropriate predictive power for all endogenous variables. Table 4 summarizes the assessment results of the measurement model. All 12 hypotheses are supported.

**TABLE 4:** Parameter estimation and hypothesis testing of the analysis model.

Path	Estimated value	Standard error	*P*-value	Hypothesis
Bonding social capital←Sports events	0.191	0.056	**	H1a surpported
Bridging social capital←Sports events	0.120	0.058	*	H1b surpported
Team identity←Bonding social capital	0.373	0.048	***	H2a surpported
City identity←Bonding social capital	0.237	0.057	***	H2b surpported
Team identity←Bridging social capital	0.418	0.048	***	H3a surpported
City identity←Bridging social capital	0.238	0.058	***	H3b surpported
Event excitement←Team identity	0.447	0.050	***	H4a surpported
City attachment←Team identity	0.376	0.053	**	H4b surpported
City pride←Team identity	0.490	0.046	***	H4c surpported
Event excitement←City identity	0.170	0.054	**	H5a surpported
City attachment←City identity	0.202	0.055	***	H5b surpported
City pride←City identity	0.210	0.051	***	H5c surpported

Fit index: RMSEA = 0.061; CFI = 0.944;TLI = 0.935; χ2/df = 642.946/304 = 2.115.

*P < 0.05,**P < 0.01,***P < 0.001.

The analysis results of sports events and social capital show that the path coefficients of sports events for bonding and bridging social capital are 0.191 and 0.120 (significant at 0.05), respectively, indicating that holding sports events can positively impact social capital. Thus, H1a and H1b are empirically supported.

The analysis results of social capital and social identity show that the path coefficients of bonding social capital for team and city identity are 0.373 and 0.237 (significant at 0.001), respectively, indicating that bonding social capital can positively impact social identity. Therefore, both H2a and H2b are supported. The path coefficients of bridging social capital for team and city identity are 0.418 and 0.238 (significant at 0.001), respectively, indicating that bridging social capital can positively influence social identity. Both H3a and H3b are supported by empirical evidence.

The analysis results of social identity and psychic income show that the path coefficients of team identity for excitement, city attachment, and pride are 0.447, 0.376 and 0.490, respectively. In addition, the path coefficients are significant at 0.01, indicating that team identity has a positive impact on excitement, city attachment, and city pride. Hence, H4a, H4b, and H4c are supported. The path coefficients of city identity for excitement, city attachment, and city pride are 0.170, 0.202, and 0.210, respectively. The path coefficients are all significant at 0.01, indicating that city identity can positively affect excitement, city attachment, and city pride. Thus, H5a, H5b and H5c are all supported.

To sum up, this study used SEM to analyze the relationship between social capital, social identity, and psychic income of residents in the host city after sports events. The results revealed that social capital had a significant positive impact on social identity. Moreover, social identity significantly impacted the excitement, city attachment, and city pride in psychic income.

## Discussion

### Sports events and social capital

Bonding social capital and bridging social capital are improved after sports events, which confirms the research by Peachey et al. (2015a) and Schulenkorf (2013), indicating that sports events can change residents’ perception of their social networks and thus improve social capital. Qingdao Marathon has attracted many local residents to participate. During the participation, local residents strengthen their communication with each other, and their behaviors and values also influence each other (Misener and Mason, 2006). The marathon event enriches the sports and cultural life of local residents, enhances their quality, and improves interpersonal relations. By strengthening the connection between residents, small groups may even be formed to improve social capital, which is conducive to maintaining social stability in the long run.

### Social capital and social identity

Social capital shows a significant positive impact on social identity after sports events. Through social capital, residents form a smaller, more unique group. The formation of this group can strengthen social identity (Woolf et al., 2013). Large sports events can attract widespread social concerns, and a marathon event in particular can attract many local residents to participate or watch the game together. Through verbal interactions during the Qingdao marathon, the residents’ sense of unity, friendship, and communication are promoted. In turn, the social capital of residents is also improved, further enhancing social identity, which supports the findings of Sherry et al. (2015). During the event, residents can feel the efforts achieved by the whole city in urban management and construction. Residents’ understanding of city managers is improved, and the connection between residents and the city is closed, strengthening city identity. In addition, many residents are fanatical about the local team after watching the game and even become fans, and thus strengthening team identity, which supports the research by Fredline (2004).

### Social identity and psychic income

After sports events, residents can identify with their city or the team they support through social capital to generate psychic income. This result supports the findings of Clopton and Finch, 2010 that individuals must first be able to generate connections (i.e., social capital), and then identity (i.e., social identity) can be socially anchored, thus generating psychic income. Therefore, psychic income cannot be realized only by strengthening the connection between residents. Residents should feel the cognitive connection with society and identify with it before psychic income can be realized.

In the structure of psychic income, the influence of social identity on event excitement is established, proving that the excitement and enthusiasm of residents can be aroused during the event, i.e., sports events create excitement for urban residents (Anderson and Holden, 2008). The path coefficient of event excitement is the lowest, indicating that the impact of the event dwindles and residents in the host city becomes calmer after the event, which supports the temporality of psychic income (Schulenkorf, 2010). In terms of city attachment, although individuals return to daily life after the event, the collectivity and social connection of residents are improved, and their sense of belonging to the city is improved based on the long-term potential of social identity. These findings support the relevant research by Kim and Walker (2012); Kim et al. (2015). The timing of city attachment can transcend the effect of sports events. In addition, according to the psychic continuum model (Funk and James, 2001), the initial consciousness of consumers affects their potential loyalty. It can be inferred that for sports events, residents are likely to be attracted by events in some circumstances and gradually evolve into attachment or loyalty to the host city, which confirms the research result suggested by Shapiro et al. (2012). In terms of city pride, individuals can temporarily immerse in sports events in an atmosphere of competition and cooperation, free from the influence of social pressure, and feel that sports events are interesting and pleasant, thus generating pride. This result supports the previous findings suggested by Heere et al. (2013). The entry threshold of marathon events is not high, and many people are usually attracted around the route, which can fully mobilize the enthusiasm of residents to watch the race and form a mass sports event. Therefore, residents in the host city can generate a strong sense of city pride.

## Conclusion

Based on social capital and social identity theories, this study explores the impact of holding sports events on the psychic income of residents. The results reveal that a marathon event can improve their psychic income through social capital and social identity. Future researchers and practitioners can expand the sample size. Based on the research results, longitudinal studies can be conducted on other potential intangible social and emotional benefits brought by sports events to explore the dynamic mechanism of their relationship and the positive impact of sports events.

## Data availability statement

The raw data supporting the conclusions of this article will be made available by the authors, without undue reservation.

## Ethics statement

Ethical review and approval was not required for the study on human participants in accordance with the local legislation and institutional requirements. Written informed consent from the patients/participants or patients/participants legal guardian/next of kin was not required to participate in this study in accordance with the national legislation and the institutional requirements.

## Author contributions

The author confirms being the sole contributor of this work and has approved it for publication.

## Conflict of interest

The author declares that the research was conducted in the absence of any commercial or financial relationships that could be construed as a potential conflict of interest.

## Publisher’s note

All claims expressed in this article are solely those of the authors and do not necessarily represent those of their affiliated organizations, or those of the publisher, the editors and the reviewers. Any product that may be evaluated in this article, or claim that may be made by its manufacturer, is not guaranteed or endorsed by the publisher.
